# A cysteine near the C-terminus of UCH-L1 is dispensable for catalytic activity but is required to promote AKT phosphorylation, eIF4F assembly, and malignant B-cell survival

**DOI:** 10.1038/s41420-019-0231-1

**Published:** 2019-12-10

**Authors:** Sajjad Hussain, Tibor Bedekovics, Asma Ali, Omar Zaid, Danielle G. May, Kyle J. Roux, Paul J. Galardy

**Affiliations:** 1 Department of Pediatric and Adolescent Medicine, Mayo Clinic, Rochester, MN 55905 USA; 2 Enabling Technology Group, Sanford Research, Sioux Falls, SD 57104 USA; 3 Department of Pediatrics, Sanford School of Medicine, University of South Dakota, Sioux Falls, SD 57105 USA; 4 Department of Biochemistry and Molecular Biology, Mayo Clinic, Rochester, MN 55905 USA; 5 Division of Pediatric Hematology-Oncology, Mayo Clinic, Rochester, MN 55905 USA

**Keywords:** B-cell lymphoma, Cell death, Nutrient signalling, Oncogenes, Myeloma

## Abstract

The enzyme UCH-L1 is a neuro-endocrine and germinal center B-cell marker that contributes to the development and aggressive behavior of mature B-cell malignancies. While mutations in this enzyme have been associated with Parkinson’s disease, relatively little is known about the molecular features associated with the biochemical activities of UCH-L1. Here we use a survival-based complementation assay and site-directed mutagenesis and identify a novel role for the C-terminus of UCH-L1 in supporting cell survival. The C220 residue is required for UCH-L1 to promote the assembly of mTOR complex 2 and phosphorylation of the pro-survival kinase AKT. While this residue was previously described as a potential farnesylation site, destruction of the putative CAAX motif by adding a C-terminal epitope tag did not interfere with cell survival, indicating an alternate mechanism. We used proximity-based proteomics comparing the proteomes of wild-type and C220S UCH-L1 and identified a selective loss of association with RNA-binding proteins including components of the translation initiation machinery. As a consequence, the C220S mutant did not promote the assembly of the eIF4F complex. These data identify a novel role for the C-terminus of UCH-L1 in supporting pro-survival and metabolic activities in malignant B-cells. This finding may lead to the development of therapeutics with selective activity towards malignancy that potentially avoid neuronal toxicities.

## Introduction

The biosynthesis of macromolecules is tightly regulated and is necessarily upregulated in malignant cells to support enhanced proliferation. The mTOR signaling pathway is essential in the coordinated regulation of macromolecule biosynthesis in response to nutrient availability and is frequently upregulated in malignancy^1^. The phosphorylation of the eIF4E binding protein 1 (4EBP1) by mTOR complex 1 (mTORC1) is a critical step by which mTOR promotes protein synthesis by enhancing cap-dependent mRNA translation^2^. When in its un-modified state, 4EBP1 binds to eIF4E and prevents the binding with eIF4G that is required to form the eIF4F mRNA cap-binding complex. Phosphorylation of 4EBP1 interferes with this binding allowing eIF4F assembly to occur. By reducing the non-degradative ubiquitination of raptor, the de-ubiquitinase UCH-L1 destabilizes mTORC1 leading to decreased 4EBP1 phosphorylation^3,4^. We recently described a novel mechanism by which UCH-L1 bypasses the inhibitory effect of 4EBP1 by directly associating with and promoting the assembly of eIF4F despite elevated levels of non-phosphorylated 4EBP1^3^. While this effect requires the catalytic activity of UCH-L1, the molecular events in this process are unclear.

To further understand the mechanism by which UCH-L1 regulates malignant processes, we generated a series of site-directed mutants to probe the potential roles of key residues of UCH-L1 in supporting cell survival. We have uncovered that a cysteine near the C-terminus of UCH-L1 is required to support the survival of malignant B-cells. Previously described as a site of farnesylation^5^, we find that mutation of this residue does not interfere with catalytic activity but prevents UCH-L1 from removing ubiquitin from raptor, promoting mTORC2 assembly and AKT phosphorylation and is required for its role in promoting the assembly of the eIF4F translation initiation complex.

## Results

### Site-directed mutagenesis of UCH-L1 reveals key features required to support cell survival in myeloma cells

**Fig. 1:**
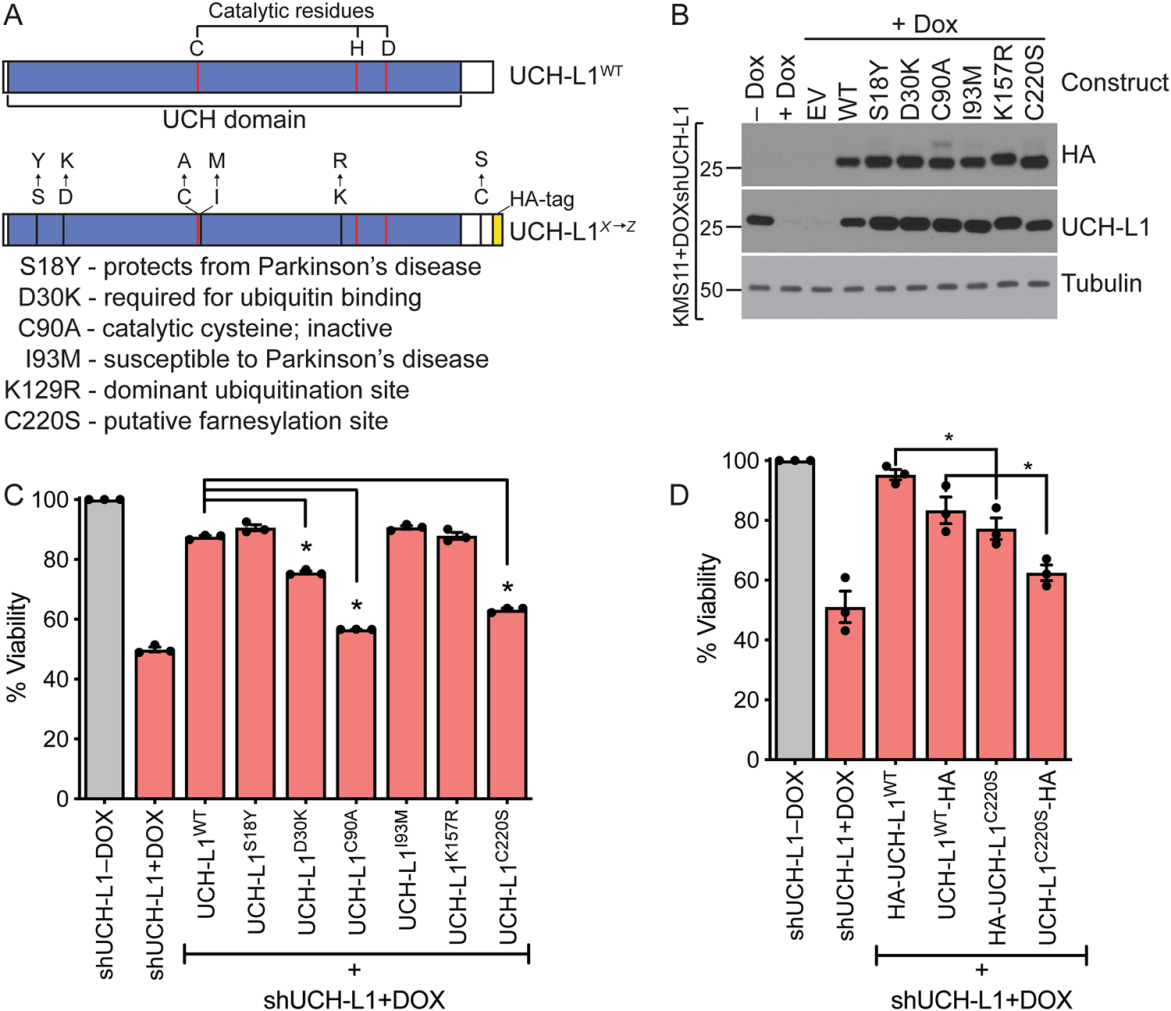
Design and expression of UCH-L1 mutants. **a** Schematic displaying the location and putative functions of the mutations studied. The residues comprising the catalytic triad are noted and further indicated by the red lines. **b** Expression of the various mutants in KMS11 myeloma cells stably transduced with a doxycycline-inducible shRNA that targets UCHL1. The blots represent typical results seen in 3–5 independent experiments. **c** Relative myeloma cell viability was determine in KMS-11 cells stably transduced with a previously characterized doxycycline-inducible shRNA in the presence or absence of the indicated UCH-L1 mutant constructs. **d** The impact of the location of the epitope tag was determined in viability assays as in **c**. The location of the tag is indicated by the order of its inclusion in the legend. The graphs in **c**, **d** represent the mean ± SEM of three independent experiments, with each point the mean of triplicates. Data indicated with an asterisk have a *p* < 0.05 using the students *t*-test.

To further explore the mechanism of how UCH-L1 contributes to B-cell malignancy, we took advantage of an shRNA-based complementation assay. KMS11 myeloma cells were stably transduced with a previously characterized doxycycline-inducible shRNA-targeting *UCHL1* to deplete endogenous protein^3,4,6–8^. These cells were then additionally transduced to express one of six shRNA-resistant mutants designed to probe the involvement of selected residues in promoting cell survival. These mutants (Fig. 1a) were designed modeled on reports of their involvement in the pathogenesis of Parkinson disease (S18Y, I93M)^9^, requirement for catalytic activity or ubiquitin binding (C90A, D30K)^10^ the dominant site for mono-ubiquitination (K129R)^11^, and a C-terminal cysteine proposed to be a site of farnesylation (C220S)^5^. All mutants were expressed at similar levels that is close to the baseline level of UCH-L1 in these cells (Fig. 1b). After depleting endogenous UCH-L1 by adding doxycycline, we monitored cell viability using MTS viability assays and compared the survival of cells expressing the mutants to control empty vector transduced cells (Fig. 1c). As expected, the expression of wild-type UCH-L1 was able to restore cell viability whereas the catalytic mutant (C90A) was unable to do so. Similar levels of cell viability were observed in cells transduced with UCH-L1 mutants associated with Parkinson’s disease (S18Y and I93M), as well as the K157R mutant, indicating that these residues do not play an important role in malignant B-cell survival. In addition to the catalytic cysteine mutant, there was a reduction in cell viability in cells transduced with the D30K mutant and a more substantial reduction in survival in cells expressing the C220S mutant.

Up to 50% of UCH-L1 is associated with cell membranes—particularly in neurons^5,12^. We have also observed that UCH-L1 associates with membrane fractions in a nutrient sensitive manner as it interacts with the mTOR signaling network^4^. The C220 residue has been identified to be a component of an atypical farnesylation motif, and it has been proposed that the farnesyl group may be responsible for membrane association^5^. Because a C-terminal epitope tag destroys the proposed farnesylation motif that must include a cysteine four residues from the C-terminus, we generated wild-type and C220S mutants with the HA tag on the N-terminus and we again found similar pattern of cell viability (Fig. 1d).

**Fig. 2:**
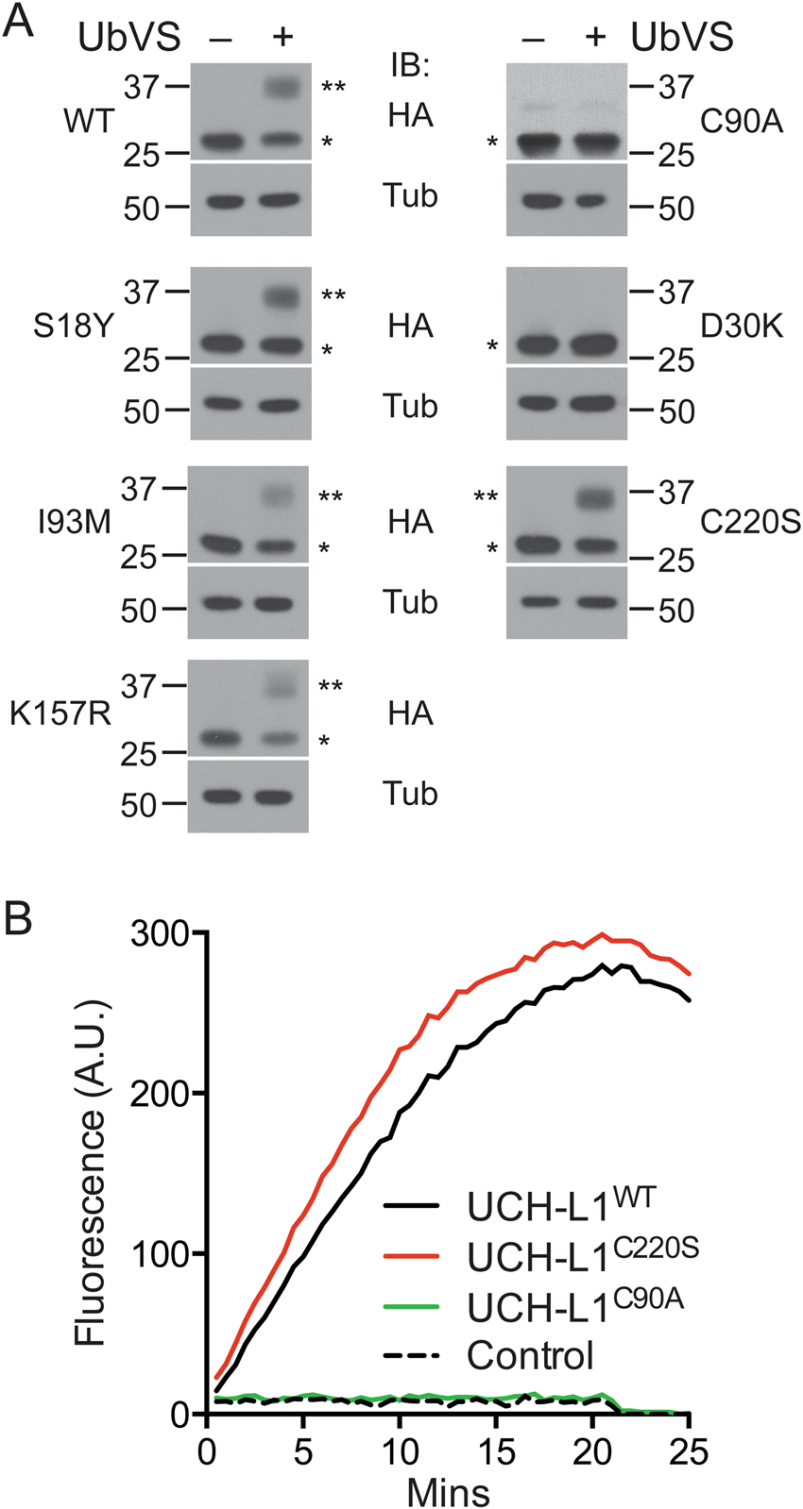
UCH-L1 catalytic activity and cysteine 220 are required for myeloma cell survival. **a** Qualitative assay reflecting catalytic activity as reflected by adduct formation with ubiquitin vinylsuflone (UbVS). Catalytic activity is reflected in a mass-shift reflecting a covalent adduct between UCH-L1 (and mutants) and UbVS as indicated. All constructs included a C-terminal HA tag. The blots represent typical results from three independent experiments. *UCH-L1, **UCH-L1-Ub-VS adduct. Tubulin (Tub) is included as a loading control. **b** Hydrolysis of the model substrate ubiquitin AMC. HeLa cells were transduced with the indicated HA-tagged UCH-L1 constructs and the proteins were purified using the epitope tag (see methods). Purified enzyme was mixed with UbAMC and the catalytic activity was monitored by the release of fluorescence from the liberated AMC moiety. The graph represents the data from one experiment that is representative of three independent experiments.

We next examined the catalytic activity of the mutant enzymes using the activity-based probe ubiquitin vinylsulfone (UbVS; Fig. 2a)^13^. Active deubiquitinating enzymes form a covalent adduct with UbVS in an activity-dependent manner resulting in an electrophoretic mass-shift detected by immunoblotting^13,14^. All constructs, with the exception of the catalytic mutant C90A and the ubiquitin binding mutant D30K, showed a similar level of adduct formation with UbVS. The lack of adduct formation with these constructs indicates catalytic inactivity of these mutants, and explains their inability to support cell survival. Because the C220S mutant has qualitatively normal activity yet cannot support cell survival, we performed a quantitative analysis of activity using ubiquitin 7-amino-4-methylcoumarin (Ub-AMC)^15^ to exclude a more subtle catalytic defect. We again found no significant difference in activity compared with that of the wild-type enzyme (Fig. 2b). These data confirm that UCH-L1 catalytic activity is required but not sufficient to support survival in KMS11 myeloma cells. The data also indicate that a non-catalytic function of UCH-L1 contributes to its role in malignant cell survival.

### C220 is required to promote mTOR-AKT signaling

**Fig. 3:**
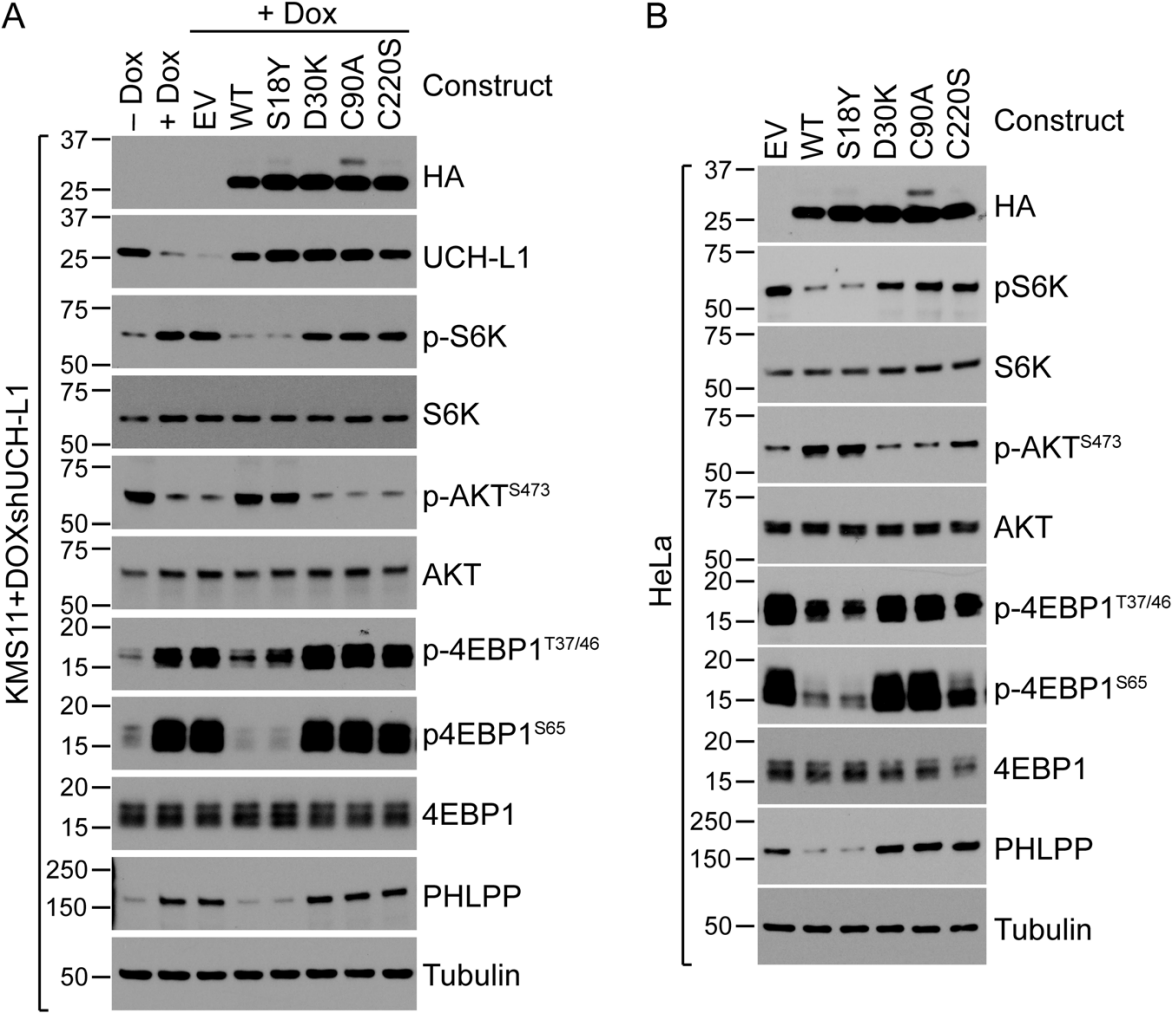
The mTOR regulating activity of UCH-L1 requires catalytic activity and C220. **a**, **b** UCH-L1 was depleted in KMS11 cells (A) stably transduced with a doxycycline-inducible shRNA-targeting UCH-L1. These cells, and HeLa cells (B) were stably transduced with the indicated C-terminal HA-tagged mutant constructs, and extracts were subjected to immunoblots with the indicated antibodies. These immunoblots represent the typical results seen in 3–5 independent experiments.

**Fig. 4:**
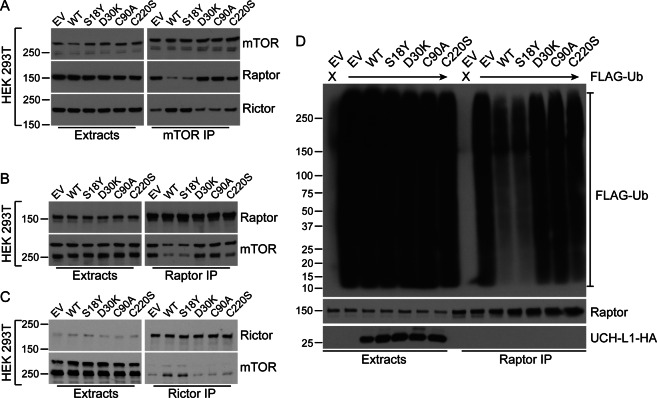
C220 is required for the suppression of raptor ubiquitination, and reorganize mTOR complex assembly. **a**–**c** Cells were transduced with empty control lentivirus or the various UCH-L1 mutants and extracts were subjected to immunoprecipitation for mTOR (**a**), raptor (**b**), or rictor (**c**), and were probed as indicated. Note how the expression of wild-type or S18Y UCH-L1 reduced the association of raptor with mTOR while increasing that of rictor while C220S, along with the catalytically inactive D30K and C90A constructs, are unable to do so. **d** Cells as in (A-C) were further transduced to express FLAG-tagged ubiquitin (FLAG-Ub) and SDS-extracts were subjected to immunoprecipitation for raptor. All blots are representative of the results in 3–5 independent experiments.

We have previously shown that UCH-L1 promotes AKT signaling by shifting the relative balance of mTOR complexes in favor of the rictor containing mTOR complex 2 (mTORC2) by suppressing the non-degradative ubiquitination of the mTORC1 subunit raptor^4^. We also previously observed that expression of a constitutively active AKT construct significantly rescues cell death induced by UCH-L1 depletion^8^. This led us to hypothesize that the UCH-L1 C220S mutant may be unable to regulate mTOR signaling. As we previously found, expression of wild-type UCH-L1 inhibits the mTORC1 phosphorylation of p70 S6 kinase (S6K) and the eIF4E binding protein 1 (4EBP1) and increases the phosphorylation of AKT^S473^ (Fig. 3a, b). The catalytically active S18Y mutant has similar effects. In contrast, the C220S mutant was largely unable to promote these changes and was similar to the catalytically inactive C90A and D30K constructs. There was a small impact of the C220S mutant on AKT phosphorylation in most experiments and this may explain why it has a slight ability to rescue cell survival. We also examined the relative levels of raptor (mTORC1) and rictor (mTORC2) containing mTOR complexes and found again that the C220S mutant is similar to the catalytically inactive C90A and D30K constructs in that it did not alter the relative amounts of mTORC1/2 (Fig. 4a–c). In agreement with this, the ubiquitination of raptor was similar with the catalytically active wild-type and S18Y constructs strongly suppressing it while the C220S mutant does not (Fig. 4d). These data indicate that despite its catalytic activity, the UCH-L1 C220S mutant is unable to deubiquitinate raptor or promote mTORC2 assembly and AKT phosphorylation.

### Proximity-based proteomics identifies UCHL1^C220^ is required for association with the eIF4F translation initiation complex

**Fig. 5:**
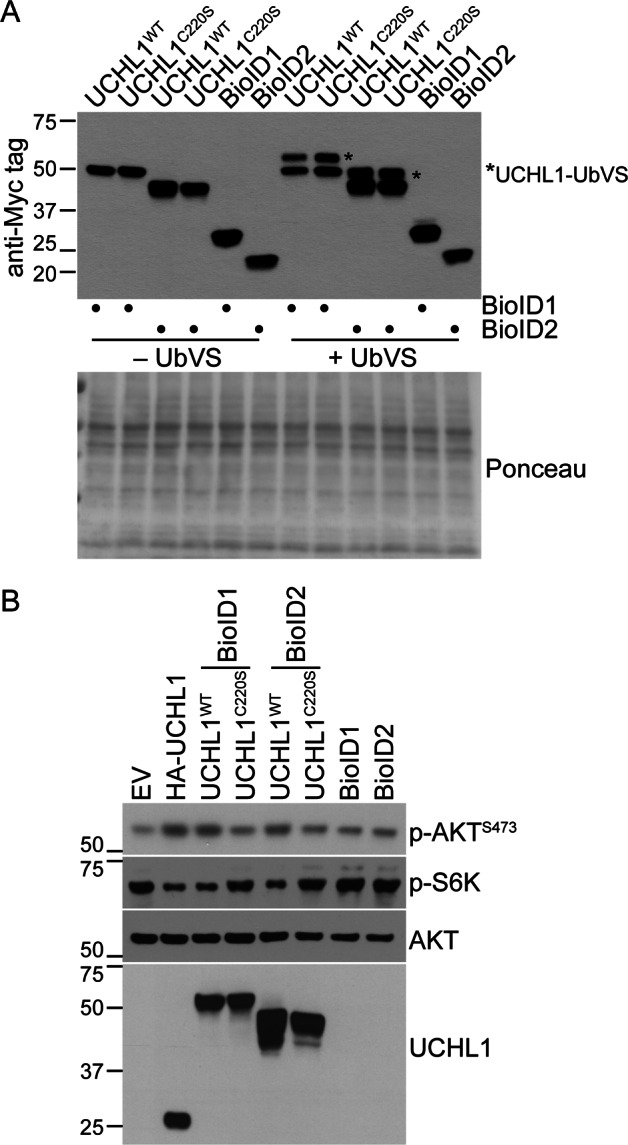
Proximity-based proteomics identifies the association of UCH-L1 with translation initiation factors. **a** Catalytic activity of the indicated fusions with BioID1 or BioID2 was assessed by reactivity with the activity-based probe ubiquitin vinylsulfone (UbVS). Activity is indicated by a shift in mass of approximately 8–10 kDa due to the covalent adduct formed with active enzymes and UbVS. Ponceau membrane staining is included as a loading control. **b** The functional integrity of the BioID fusion proteins was assessed by its ability to promote phosphorylation of AKT^S473^ and to suppress that of S6 kinase. Expression of HA-UCHL1 was used for comparison. The blots in **a** and **b** represent the typical results from 3–5 individual experiments.

We recently used a proximity-based proteomic approach to show that UCH-L1 associates with and promotes the assembly of the eIF4F complex^3^. We therefore reasoned that this technique may help to identify differences in the complexes formed by the wild-type and C220S mutant forms of UCH-L1. As we did for the wild-type UCH-L1, we generated an N-terminal fusions of the BioID1 and BioID2 biotin ligases that behave similarly in proteomic assays but differ by mass^3,16,17^. We observed a similar expression and catalytic activity with all fusions and selected the BioID2 constructs for further experimentation because of the smaller mass (Fig. 5a, b). We performed duplicate large-scale experiments involving biotinylation, streptavidin retrieval, and proteomic identification of the retrieved proteins as we previously described. A total of 241 proteins were enriched in pulldowns from cells expressing BioID2-UCHL1^C220S^ compared to those performed from cells expressing BioID2 alone, including 131 proteins that were retrieved uniquely from cells expressing BioID2-UCHL1^C220S^ (Table 1; Table 1S). We analyzed the list of unique and enriched proteins using the Database for Annotation, Visualization, and Integrated Discovery (DAVID; https://david.ncifcrf.gov/). There were three clusters with significant representation taking multiple comparisons into account, including cell–cell adhesion, RNA binding, and RNA splicing (Table 2). Notably, the cluster representing translation initiation and DEAD box helicase that were enriched in experiments with wild-type UCH-L1 were not significantly enriched in those with BioID2-UCHL1^C220S^. There were 51 proteins that were enriched at least three-fold in the wild-type experiments over the C220 mutant (Table 2S). Analyzing these through DAVID yielded only one significant cluster representing RNA-binding (Table 3). These data suggest that while the C220S mutant continues to have some association with RNA-binding proteins, there is a significant reduction compared with the wild-type enzyme.

**Table 1:** Classification of proteins retrieved by proximity biotinylation.

Identified proteins	*n*
Unique to BioID2-UCHL1C220S	131
BioID2-UCHL1C220S/BioID2 >3	110
BioID2-UCHL1C220S/BioID2 0.3-3	346
BioID2-UCHL1C220S/BioID2 <3	131
Unique to BioID2	85
Total proteins identified^a^	803

^a^Found in both replicates from at least 1 construct

**Table 2:** DAVID Functional cluster analysis of proteins retrieved with BioID2-UCHL1C220S.

Functional cluster	*P*-value
Cell–cell adhesion	1.94E-31
RNA binding	9.38E-09
RNA-splicing	5.57E-08
Translation initiation	NS
DEAD-Box helicase	NS

*N* = 262 proteins retrieved in each of two independent experiments, ratio BioID/BioID-UCHL1-C220S > 3

**Table 3:** DAVID Functional cluster analysis of proteins enriched in BioID2-UCHL1WT over BioID2-UCHL1C220S.

Functional cluster	*P*-value
RNA binding	0.02
K-Homology domain	0.03
GTP binding	0.03
Cell division	0.03

*N* = 51 proteins, retrieved in two independent experiments (WT), ratio BioID-UCHL1-WT/BioID-UCHL1-C220S >3

**Fig. 6:**
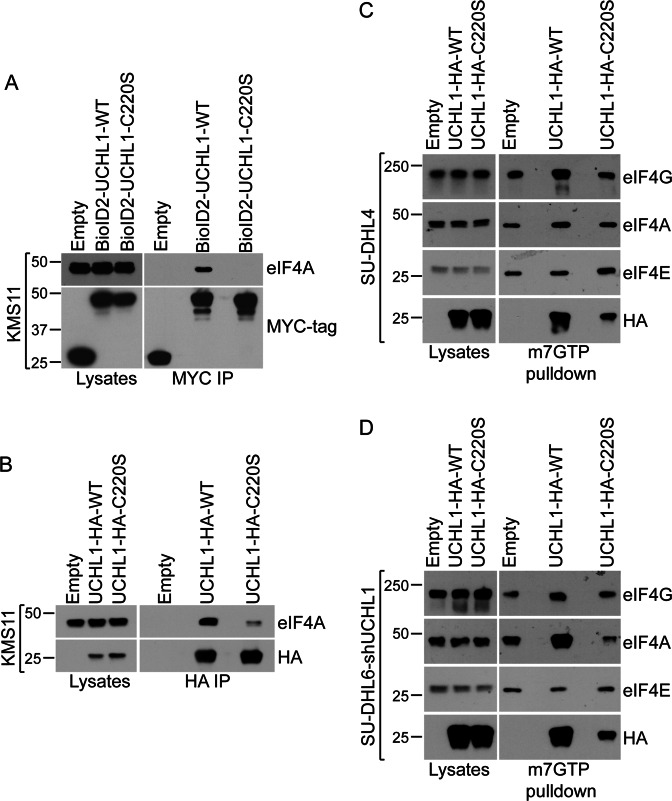
Proximity-based proteomics identifies the association of UCH-L1 with translation initiation factors. **a**, **b** KMS11 cells expressing BioID2-UCHL1 fusions (**a**) or C-terminal HA-tagged constructs were subjected to immunoprecipitation and probed as indicated. **c**, **d** UCH-L1 was introduced into non-expressing SU-DHL4 cells (**c**) or the high-expressing SU-DHL6 cells after UCH-L1 depletion (**d**) and the relative levels of the eIF4F complex was determined by pulldown with the mRNA cap analogue m7GTP coupled to agarose. The loading was normalized to the level of the cap-binding subunit eIF4E. All blots represent the typical results of 3–5 independent experiments.

Notably, the eIF4F complex subunit eIF4A was identified in the proximity proteome of wild-type UCH-L1—but was absent from the data from BioID2-UCHL1^C220S^ (Table 4). We confirmed a relative loss of eIF4A binding in pulldowns with the wild-type and C220S BioID2 constructs as well as HA-tagged constructs (Fig. 6a, b). As we previously identified that UCH-L1 promotes the assembly of the eIF4F complex, we wondered whether the C220S mutant would behave similarly. In m^7^GTP pulldowns normalized for the recovery of eI4FE (cap-binding protein) there was again an increase in the recovery of eIF4G and eIF4A from cells expressing wild-type UCH-L1, but this effect was not observed in cells expressing the C220S mutant (Fig. 6c, d). Similar results were obtained in different cell lines. This indicates that this mutation impairs the effect of UCH-L1 on the assembly of the eIF4F complex.

**Table 4:** Relative association of translation initiation factors with BioID2-UCHL1WT and BioID2-UCHL1C220S.

Gene names	Ratio C220S/BioID2	Ratio WT/C220S
EIF4A1	Not found in C220	Not found in C220
PABPC4	Infinite	18.1
EIF2S3	4.1	10.1
PABPC1	Infinite	3.9
EIF3B	1.3	2.8
EIF4G3	Infinite	1.9
EIF4G2	58.5	1.8
EIF4G1	4.3	1.5
EIF3G	Infinite	1.4
EIF5A	Infinite	1.3
EIF3H	2.6	1.2
EIF4B	22.2	1.1
ABCE1	34.9	1.1
DHX29	43.6	1.0
EIF2A	4.9	0.7

## Discussion

Aberrant high expression of UCH-L1 is seen in mature B-cell lymphoma and multiple myeloma where it promotes cell survival and predicts poor outcomes^6,7^. Depletion of UCH-L1 leads to cell death in these cancers in vitro and in an orthotopic model of myeloma in mice^6–8,18,19^. Small molecule inhibition of UCH-L1 are under development^20–23^, though the progressive neurodegeneration seen in *Uchl1* null mice^24–27^ and in humans^28^ leads some to worry that this approach may result in unacceptable neuro-toxicity. Here we describe a novel requirement for the C220 residue of UCH-L1 in supporting cell survival in malignant B-cells. Importantly, mutating this residue has no apparent impact on the catalytic activity of UCH-L1 towards two model substrates but rather interferes with its ability to promote AKT signaling and the enhanced assembly of the eIF4F translation initiation complex. We previously observed that catalytic activity was required for UCH-L1 to disrupt mTORC1, promote mTORC2 phosphorylation of AKT, and for it to promote the assembly of eIF4F^3,4,8^. The C220S mutant, therefore, is discrepant in that it is catalytically active towards model substrates but is unable to promote these biochemical changes in the mTOR-AKT and eIF4F pathways. These observations raise the potential for selective interference with oncogenic activities of this enzyme while preserving the physiologic activity to prevent neurologic symptoms. Countering this notion, however, is our prior observation that deletion of *Uchl1* in the mouse leads to a dramatic increase in mTORC1 signaling in the brain. While the C220S mutant is catalytically active, we clearly show that it is defective in suppressing mTORC1 activity. As increased brain mTORC1 activity in other contexts—such as in tuberous sclerosis—is pathogenic, interfering with the C-terminus of UCH-L1 may ultimately have a similar physiological effect. As there is no clear in vitro surrogate for the neurodegeneration seen in mice lacking UCH-L1, the answer to this question will ultimately require the generation of a *Uchl1*^C220S^ knock-in mouse model.

There is ongoing controversy regarding the role of the C220 residue as a potential farnesyl acceptor site in UCH-L1. This notion has been called into question after an observation that inhibition of farnesylation with a small molecule inhibitor or mutation did not appreciably affect the localization or membrane association of UCH-L1^12^. Furthermore, our transgenic mouse model^8^ included a hemagglutinin (HA) epitope tag on the C-terminus of UCH-L1, which destroys the farnesylation motif—indicating that this mechanism is not essential for its oncogenic activities. The biochemical role of farnesylation on the membrane association of UCH-L1 is further in question after a recent report found that mutation of C220S and other cysteine residues in UCH-L1 did not change the fraction of UCH-L1 associated with membranes^12^. This fraction did, however, increase in cells expressing a mutant in which the C-terminal four residues were removed. This property was associated with a substantial loss in catalytic activity as measured by UbVME reactivity as well as reduced solubility with partitioning of the mutant into triton X-100 insoluble fraction suggesting protein conformation change and aggregation. This leads us to speculate that the C220S mutant may have a more subtle effect on the secondary structure of UCH-L1 that does not affect catalytic activity but its ability to fully participate in protein–protein interactions. More recently, the C220 residue and farnesylation of UCH-L1 was implicated in mediating the association with the Epstein Barr Virus (EBV) latent membrane protein 1 (LMP1) and the sorting of LMP1 into exosomes^29^. Whether these findings may also relate to subtle changes in secondary structure is unclear.

Our results further reinforce the importance of the metabolic and signaling impacts of UCH-L1 on malignant cell survival. One of the most consistent findings has been that UCH-L1 associated cell survival is linked with its ability to promote AKT signaling. Our data here again find that any mutant that fails to promote this signaling change cannot support cell survival. These results suggest then that B-cell malignancies with high levels of UCH-L1 may be particularly sensitive to inhibitors that shut down signaling through the PI3K pathway, AKT itself, mTOR kinase inhibitors that would inhibit AKT phosphorylation by mTORC2. The results that further show that UCH-L1 stimulates eIF4F assembly suggest that protein synthesis inhibition may be another novel strategy in these cases.

## Materials and methods

### Cell lines, culture, generation of mutants

The myeloma cell lines KMS11 and KMS12 cell lines were kindly provided by Takemi Otsuki. HeLa, HEK293T, SU-DHL,4, and SU-DHL6 cells were obtained from the American Type Culture Collection. All cells were maintained under standard conditions. The identity of all cell lines are verified annually in our laboratory. Point mutations in UCH-L1 (Fig. 1) were prepared using the GeneArt mutagenesis kit (Invitrogen/ThermoFisher Scientific) according to the manufacturer’s instructions. UCH-L1 and mutant expression constructs were cloned into the TSiN lentiviral vector as previously described. Depletion of UCH-L1 was performed with a pTRIPZ-based doxycycline-inducible shRNA-targeting UCH-L1 as previously characterized and described. The generation of stable, pooled, transduced cells has been previously described. To prevent loss of transgene or shRNA expression, transduced cells are re-derived on a regular basis and the expression and depletion of relevant targets is monitored during each experiment.

### Ubiquitin hydrolase activity assays

Qualitative catalytic activity of wild-type and UCH-L1 mutant constructs was assayed using the activity-based probe ubiquitin vinylsulfone (Boston Biochem, Cambridge, MA) as previously described. Quantitative catalytic activity for purified wild-type and C220S mutants was assayed using the fluorogenic substrate ubiquitin-AMC (Boston Biochem) using a molecular devices automated plate reader. The reaction was conducted in a buffer containing 50 mM HEPES, 0.5 mM EDTA, 1 mg/ml ovalbumin, 1 mM DTT, 100 nM UbAMC, pH 7.8. The final concentration of the UCH-L1 mutants is unknown, but equal volumes of enzyme are added from stocks with equal (by SDS-PAGE) amounts of protein. Proteins were purified using a small-scale HA-purification system (MBL International, Woburn, MA) from cells expressing the respective constructs.

### Immunoreagents, immunoprecipitation, m7GTP pulldown assays

Antibodies used include UCH-L1 (3524), raptor (2280), rictor (9476), 4EBP1 (2845), p4EBP1^T70^ (5078), p4EBP1^T37/46^ (2855), p4EBP1^S65^ (9451), pS6K (9208), S6K (49D7), AKT (4691), pAKT^S473^ (4060), eIF4A (C32B4), eIF4E (C46H6), anti-DYKDDDDK Tag (8146), anti-MYC tag (9B11) from Cell Signaling Inc. (Danvers, MA, USA), anti-HA (3F10, Roche Applied Science, Indianapolis, IN, USA) tubulin (T9026) from Sigma, mTOR (A301-143a), PHLPP (A300-660A) from Bethyl Laboratories (Montgomery, TX, USA), eIF4G (ab31217) from Abcam. Immunoprecipitations were performed as described using lysates prepared with 0.3% CHAPS (mTOR/rictor/raptor)^4,30^, 0.5% NP40 (BioID2-UCHL1, UCHL1-HA) or 1% SDS (raptor for detection of FLAG-Ub)^4^. Immobilized m^7^GTP (AC-155) was from Jena Bioscience (Jena, Germany). M^7^GTP pulldowns were performed as described^3^.

### Proximity-based proteomics, pathway analysis

The BioID2-UCHL1^C220S^ fusion^16^ was prepared by cloning the UCH-L1^C220S^ mutant with the BioID2 cDNA fused to the N-terminus as described^3^. KMS11 cells expressing the BioID2-UCHL1^C220S^ fusion were pulsed with biotin, lysed, and the biotinylated proteins were isolated and analyzed as described^3,31^. The proteins identified were analyzed as previously described^3^ using the Database for Annotation, Visualization, and Integrated Discovery (DAVID) web tool (https://david.ncifcrf.gov), using the default DAVID annotation categories, with the classification stringency set to high. The terms within each retrieved annotation cluster were summarized, and a mean *P*-value was calculated.

## Supplementary information

Table 1S

Table 2S
